# Factors affecting the topography of nitrous oxide‐induced neurological complications

**DOI:** 10.1111/ene.16291

**Published:** 2024-03-26

**Authors:** Eva Sole Cruz, Etienne Fortanier, Frederic Hilezian, Adil Maarouf, Clémence Boutière, Sarah Demortière, Audrey Rico, Emilien Delmont, Jean Pelletier, Shahram Attarian, Bertrand Audoin

**Affiliations:** ^1^ Reference Center for Neuromuscular Diseases and ALS La Timone University Hospital, APHM Marseille France; ^2^ APHM, Department of Neurology La Timone University Hospital, APHM Marseille France; ^3^ Aix‐Marseille University, CRMBM UMR 7339, CNRS Marseille France; ^4^ Aix‐Marseille University, INSERM, GMGF Marseille France

**Keywords:** nitrous oxide, neuropathy, myelopathy

## Abstract

**Background:**

The factors underlying the topography of nitrous oxide (N_2_O)‐induced neurological complications are unknown.

**Methods:**

We included all consecutive patients admitted to the university hospital of Marseille for N_2_O‐induced neurological complications in a prospective observational study. Patients underwent neurological examination, spinal cord magnetic resonance imaging, and nerve conduction studies within the first 4 weeks after admission.

**Results:**

In total, 61 patients were included: 45% with myeloneuropathy, 34% with isolated myelopathy, and 21% with isolated neuropathy. On multivariable analysis, the odds of myelopathy were associated with the amount of weekly N_2_O consumption (~600 g cylinder per week, odds ratio [OR] = 1.11, 95% confidence interval [CI] = 1.001–1.24). The extent of the myelopathy (number of vertebral segments) was correlated with the number of ~600‐g cylinders consumed weekly (ρ = 0.40, *p* < 0.005). The odds of neuropathy were associated with the duration of consumption (per month; OR = 1.29, 95% CI = 1.05–1.58). Mean lower‐limb motor nerve amplitude was correlated with the duration of consumption (in months; ρ = −0.34, *p* < 0.05).

**Conclusions:**

The odds of myelopathy increased with the amount of N_2_O consumption, and the odds of neuropathy increased with the duration of N_2_O exposure, which suggests distinct pathophysiological mechanisms underlying these two neurological complications.

## INTRODUCTION

In the past few years, the heavy misuse of nitrous oxide (N_2_O) as a recreational drug has led to the admission in the emergency departments of numerous patients presenting acute or subacute sensorimotor deficits due to axonal polyneuropathy and/or myelopathy [1, 2, 3, 4, 5, 6, 7, 8, 9]. However, little is known about the factors affecting the topography and severity of nervous system injuries associated with N_2_O abuse and, in particular, why some patients predominantly present spinal cord involvement and others peripheral neuropathy.

In 2020, we decided to harmonize our practice for all patients admitted in the university hospital of Marseille for N_2_O‐induced neurological complications. Particularly, we planned to perform spinal cord magnetic resonance imaging (MRI) and biological and electrophysiological explorations for all patients, associated with an extensive clinical assessment, within the first 4 weeks after admission. The aim of the study was to determine the factors affecting the topography and severity of nervous system complications associated with the recreational use of N_2_O.

## METHODS

### Study design

Since 2020, we included all consecutive patients admitted for N_2_O‐induced neurological complications in a prospective observational study.

The amount (in ~600‐g cylinders) and duration of N_2_O consumption were collected for all patients, along with other toxic habits. Clinical scores included the motor Medical Research Council sum score, the modified Inflammatory Neuropathy Cause and Treatment sensory sum score (without the 2‐point discrimination item), the Overall Neuropathy Limitations Scale score (ONLS), the Romberg score, and the modified Rankin Scale (mRS) disability score. We measured serum levels of vitamin B12, homocysteine, and methylmalonic acid at admission before any supplementation. All spinal cord MRI examinations were performed in the same magnetic resonance unit. Electrophysiological testing was performed with a Viking Select, Viasys, v10b (2002) system or a NATUS Dantec Keypoint 2.32 (2015) system. Nerve conduction studies (NCSs) assessed eight motor nerves (median, ulnar, tibial, and peroneal nerves on both sides) and eight sensory nerves (median, ulnar, sural, and superficial fibular nerves on both sides). Needle electromyographic study included at least four muscles, two in the upper limbs and two in the lower limbs. Axonal polyneuropathy was defined as reduced amplitudes of sensory and/or motor action potentials (<80% of lower limit of normal) in at least two nerves, without demyelinating features.

### Statistical analysis

We used JMP Pro 16.1.0 (SAS Institute) for statistical analyses. The Mann–Whitney test or Fisher exact test was used for between‐group comparisons and Spearman rank correlation test for correlation analysis. For descriptive analysis, we divided patients into three groups: patients with isolated myelopathy, isolated neuropathy, or myeloneuropathy.

Univariable and multiple linear regression analyses were used to assess factors associated with the occurrence of myelopathy and neuropathy. Variables tested were age, sex, consumption of N_2_O in ~600‐g cylinders/week, duration of N_2_O consumption in months, and body mass index. Univariable and multiple linear regression analyses were used to assess factors associated with an mRS score > 2. Variables tested were age, sex, presence of myelopathy, and presence of neuropathy.

### Standard protocol approvals, registrations, and patient consents

The study was approved by the institutional review board of the university hospital of Marseille, France (reference no. RGPD 2019–01 PADS22‐396).

## RESULTS

### Study population

In total, 61 patients were included in the study (Table 1). The mean (SD) time since first N_2_O consumption and first symptoms was 9 (7) months. At admission, clinical scores, spinal cord MRI findings, serum vitamin B12 levels, and homocysteine findings were available for all patients. NCS findings were available for 53 patients (87%) and serum methylmalonic acid findings for 44 (72%). Among patients with spinal cord MRI and NCS explorations (*n* = 53), 24 (45%) presented myeloneuropathy, 18 (34%) isolated myelopathy, and 11 isolated neuropathy (21%).

Regarding electrophysiological characteristics, only one patient displayed demyelinating features with conduction blocks in both peroneal nerves below the fibula entrapment site. All the other NCS explorations were purely axonal.

**TABLE 1:** Characteristics of patients.

Characteristic	Patients with myeloneuropathy (MN; *n* = 24)	Patients with isolated myelopathy (M; *n* = 18)	Patients with isolated neuropathy (N; *n* = 11)	*p*
Age, years, mean (SD)	23 (4.5)	26 (5)	24 (5)	MN vs. M = 0.06 MN vs. N = 0.52 M vs. N = 0.40
Sex, F/M, *n*	9/15	7/11	4/7	MN vs. M = 1 MN vs. N = 1 M vs. N = 1
BMI, kg/m^2^, mean (SD)	24.5 (7)	29 (9)	27 (4.5)	MN vs. M = 0.15 MN vs. N = 0.31 M vs. N = 0.98
N_2_O consumption				
Weekly N_2_O amount in ~600‐g cylinders, median (range)	19 (2–56)	6 (1–35)	4 (0.5–21)	MN vs. M = 0.04 MN vs. N = 0.01 M vs. N = 0.41
Total N_2_O exposure time, months, median (range)	10 (1–36)	6 (1–12)	12 (6–24)	MN vs. M = 0.06 MN vs. N = 0.33 M vs. N = 0.007
Blood testing				
Vitamin B12 level, pmol/L, mean (SD) (normal values > 200 pmol/L)	168 (67)	178 (73)	203 (107)	MN vs. M = 0.81 MN vs. N = 0.48 M vs. N = 0.77
Homocysteine level, μmol/L, mean (SD) (normal values < 14 μmol/L)	103 (47)	86 (37)	66 (38)	MN vs. M = 1 MN vs. N = 0.23 M vs. N = 0.70
Methylmalonic acid level, μmol/L, mean (SD)^a^ (normal values < 0.4 μmol/L)	7.5 (6.5)	5 (4)	7.5 (8)	MN vs. M = 0.29 MN vs. N = 0.91 M vs. N = 0.63
Motor findings				
MRCss, mean (SD) (/60)	54 (5)	56.5 (3)	55.5 (4)	MN vs. M = 0.12 MN vs. N = 0.43 M vs. N = 0.61
MRCss upper limbs, mean (SD) (/30)	29 (1.5)	28.5 (2)	29 (1.5)	MN vs. M = 0.59 MN vs. N = 0.68 M vs. N = 0.72
MRCss lower limbs, mean (SD) (/30)	25 (4)	28 (2.5)	26.5 (3)	MN vs. M = 0.01 MN vs. N = 0.39 M vs. N = 0.26
Sensory findings				
Modified INCAT sensory sum score, mean (SD) (/16)	8.5 (3)	10 (2.5)	7.5 (3)	MN vs. M = 0.06 MN vs. N = 0.46 M vs. N = 0.03
Romberg score, mean (SD)	2.5 (1)	2.5 (1)	2 (1)	MN vs. M = 0.91 MN vs. N = 0.12 M vs. N = 0.15
**Severity**				
Modified Rankin Scale score, mean (SD)	3.5 (1)	3 (1)	2.5 (1)	MN vs. N = 0.01 MN vs. M = 008 M vs. N = 0.43
Overall Neuropathy Limitations Scale score, mean (SD)	5 (1.5)	5 (2)	3.5 (1)	MN vs. N = 0.01 MN vs. M = 0.20 M vs. N = 0.38

Abbreviations: BMI, body mass index; F, female; INCAT, Inflammatory Neuropathy Cause and Treatment; M, male; MRCss, Medical Research Council sum score.

^a^ Available in 17 patients with myeloneuropathy, 14 patients with isolated myelopathy, and nine patients with isolated neuropathy.

A total of three (5%) patients reported frequent cocaine use, seven (11%) alcohol abuse, 16 (26%) cannabis use, 22 (36%) daily tobacco use, and 17 (29%) use of at least two of the above drugs in addition to N_2_O. Regarding extraneurological complications of N_2_O abuse, one patient presented deep vein thrombosis. None presented cognitive or pseudopsychiatric manifestations.

### Factors affecting the topography and clinical severity of nervous system N_2_O‐related injuries

**FIGURE 1:**
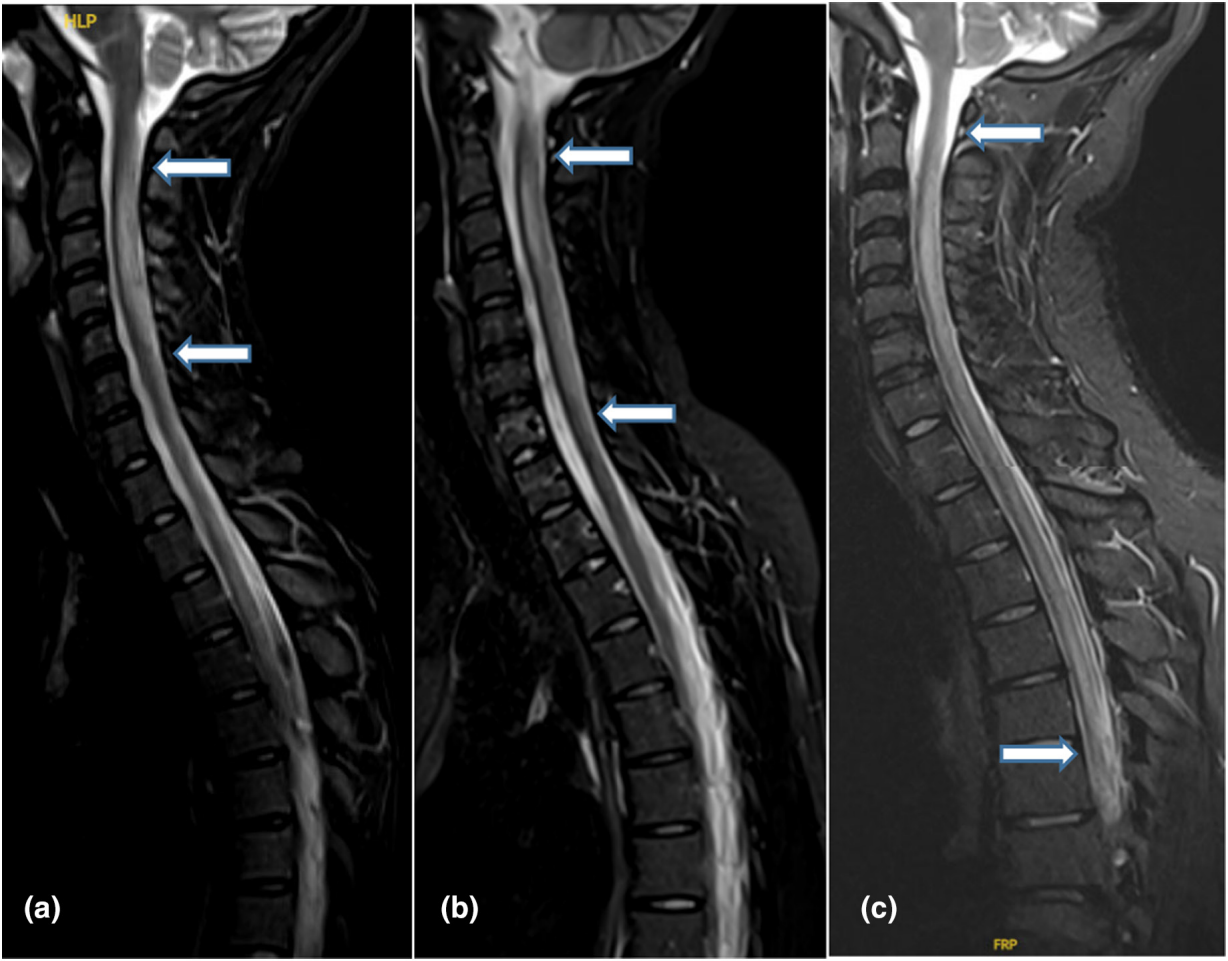
Myelitis extension according to N_2_O consumption. (a) Spinal magnetic resonance imaging (MRI; sagittal section) of a 25‐year‐old patient presenting a C2–C5 myelitis. Median weekly N_2_O consumption = 4 cylinders. (b) Spinal MRI (sagittal section) of a 22‐year‐old patient presenting a C2–C7 myelitis. Median weekly N_2_O consumption = 7 cylinders. (c) Spinal MRI (sagittal section) of a 25‐year‐old patient presenting a C2–T6 myelitis. Median weekly N_2_O consumption = 21 cylinders.

On multivariable analysis, the odds of myelopathy were associated with the amount of weekly N_2_O consumption (per ~600‐g cylinder; odds ratio [OR] = 1.11, 95% confidence interval [CI] = 1.001–1.24, *p* < 0.05). The extent of the myelopathy (number of vertebral segments) was correlated with the number of weekly ~600‐g cylinders of N_2_O consumed (Spearman ρ = 0.40, *p* < 0.005), as shown in Figure 1. On multivariable analysis, the odds of axonal neuropathy were associated with the duration of N_2_O consumption (per month; OR = 1.29, 95% CI = 1.05–1.58, *p* < 0.005). The mean of the amplitudes of the peroneal and tibial nerves was correlated with the duration of N_2_O consumption (in months; Spearman ρ = −0.34, *p* < 0.05).

On admission, the mean (SD) homocysteine level (in μmol/L) was higher for patients with than without myelopathy (98 [45] vs. 69 [38], *p* < 0.05), but vitamin B12 and methylmalonic acid levels did not differ between the groups. Levels of homocysteine, vitamin B12, and methylmalonic acid did not differ between patients with and without neuropathy.

On multivariable analysis, the odds of spinal cord involvement were associated with an mRS score > 2 at admission (OR = 7, 95% CI = 1.5–34, *p* < 0.05).

Clinical follow‐up data were available for 36 of 61 (59%) patients at 3 months. Most patients reported that they had stopped N_2_O consumption at that time. The mean (SD) ONLS improved to 1.4 (1) at 3 months (vs. 4.7 at baseline, *p* < 0.001). The mean (SD) mRS score improved to 1.5 (1) (vs. 3 [1] at baseline, *p* < 0.0001). Six of 36 (16.5%) patients had an mRS score > 2 at 3 months. On multivariable analysis, no factor was significantly associated with an mRS score > 2 at 3 months. Of note, no patient without neuropathy at baseline had an mRS score > 2 at 3 months.

## DISCUSSION

The present study found that the amount and duration of N_2_O consumption affected the topography of nervous system N_2_O‐related injuries. Involvement of the spinal cord seemed particularly dependent on the amount of N_2_O consumption, and the odds of developing axonal polyneuropathy increased with the duration of N_2_O exposure. The severity of the initial clinical deficit was mainly driven by spinal cord involvement. Finally, patients with both myelopathy and axonal polyneuropathy had the most severe clinical presentations.

The strength of the present study is its exhaustive explorations performed shortly after admission in a relatively large sample of patients. Of note, these patients were admitted via the emergency department of the university hospital of Marseille and probably reflect the most severe spectrum of N_2_O‐related neurological complications. Hence, the daily amount of N_2_O use was larger than that previously reported [1, 2, 3, 6]. This high level of consumption was probably enabled by the development in the past years of larger N_2_O cylinders of ~600 g that are easily available in numerous shops in Marseille. These new contents probably explain the increased number of patients admitted in an emergency department for severe neurological symptoms mainly related to spinal cord involvement, which is particularly sensitive to the amount of N_2_O consumption, as evidenced here. In contrast, patients with isolated axonal polyneuropathy due to a lower amount of chronic N_2_O consumption could experience more subtle clinical deficits leading less frequently to hospitalization.

The pathophysiological mechanisms underlying N_2_O‐related myelopathy and those underlying neuropathy are not fully understood. Inhibition of vitamin B12‐dependent enzyme reactions by N_2_O due to inactivation of vitamin B12 could be the main factor contributing to myelopathy [10, 11]. This functional deficiency of vitamin B12 is responsible for hyperhomocysteinemia, which could explain the higher homocysteine level in our patients with than without myelopathy. However, vitamin B12 inactivation was probably not the only cause of axonal neuropathy, because N_2_O‐induced neuropathy differs from neuropathies with chronic vitamin B12 deficiency [12, 13, 14]. Particularly, chronic hypoxia was hypothesized to contribute to axonal neuropathy [13]. N_2_O diffuses rapidly from the pulmonary circulation into the pulmonary alveoli, causing a dilution of each of the other gases and a decrease in their alveolar concentration. In the case of repeated N_2_O abuse, repeated hypoxia occurs and can cause axonal polyneuropathy, as previously described for repeated nocturnal hypoxia [15]. This situation could explain the association we found between odds of axonal polyneuropathy and duration of N_2_O consumption, as studied previously [14].

Our study was limited by its monocentric aspect, which explains the small sample size. Moreover, although a 3‐month follow‐up consultation was scheduled for each patient, 40% failed to attend, which limited the possibility of collecting follow‐up data as described in other cohorts [1].

To our knowledge, this is the first study to show that the mode of N_2_O consumption (in terms of quantity and duration) influences the clinical presentation as myelitis or peripheral neuropathy and offers different pathophysiological hypotheses underlying either presentation. Further studies are needed to understand the exact pathophysiological and toxic mechanisms induced by these different consumption patterns.

## CONFLICT OF INTEREST STATEMENT

All authors certify that there is not any financial interest related to this study.

## Data Availability

The data that support the findings of this study are available from the corresponding author upon reasonable request.
